# Molecular Simulation of the Separation of Some Amino Acid Enantiomers by β-Cyclodextrin in Gas-Phase

**DOI:** 10.3389/fchem.2020.00823

**Published:** 2020-09-08

**Authors:** Elena Alvira

**Affiliations:** Departamento de Física, Universidad de La Laguna, San Cristóbal de La Laguna, Spain

**Keywords:** cyclodextrins, amino acids, enantiomers, molecular mechanics, molecular dynamics, inclusion complex, elution order

## Abstract

The complexes formed by β-cyclodextrin and some amino acids (alanine, valine, leucine, and isoleucine) in vacuo are studied by molecular mechanics and dynamics simulations. These methods have been improved with respect to our previous studies with amino acids, regarding the determination of molecular structures or initial enantiomer dispositions in the molecular dynamics trajectories. The greatest contribution to the interaction energy is from the van der Waals term, although the discrimination between enantiomers is due mainly to the electrostatic contribution. The lowest energy structures of the complexes obtained from molecular mechanics are inclusion complexes in which the carboxylic end of amino acids is pointing toward the narrow (D-) or wide rim (L-) of β-cyclodextrin. The position probability density provided by molecular dynamics also confirms inclusion complex formation, because the guests spend most time inside the cavity of β-cyclodextrin along its axis, with the carboxylic end pointing toward the narrow rim. The L-amino acids are the first eluted enantiomers in all cases and chiral discrimination increases with the size of guests, except leucine, which has the lowest capacity to discriminate. During the simulation, Ala and Val remain in weakly enantioselective regions, while Leu and Ile stay in zones with great chiral selectivity.

## Introduction

Cyclodextrins (CDs) are macrocyclic molecules composed of glucose units (6 for α-CD, 7 for β-CD, 8 for γ-CD, etc.) forming truncated cone shaped compounds. These have cavities with different internal diameters capable of including molecules with different structure, size and composition (Szejtli and Osa, 1996; Lipkowitz, 1998, 2001). CDs are extensively used in catalysis and separation of enantiomers, which are very useful techniques in the pharmaceutical, cosmetic, chemistry and food industries (Maier et al., 2001). Amino acids are biologically important organic compounds, which in the form of proteins comprise the largest component (other than water) of human cells, muscles and other tissues. The enantiodiscrimination and inclusion complex formation of amino acids and amino-acid derivatives by CDs have been experimentally studied with different techniques such as electrospray mass spectrometry, capillary electrophoresis and gas chromatography (Ramanathan and Prokai, 1995; Boniglia et al., 2002). Experimental results also reveal the influence of organic modifiers and pH conditions on the enantiomer separation of amino acids and inclusion complex formation with CDs (Zia et al., 2001; Sebestyén et al., 2012; Stepniak et al., 2017). In particular, the chiral separation and inclusion complexes formed by valine, leucine, and isoleucine (among other amino acids, but not alanine) with partially methylated β-CD in gas-phase, have been analyzed both experimentally and theoretically (Ramirez et al., 2000). It has been established that the enantiomers can locate almost totally inside the cavity in the complexes of minimum energy, and the L-amino acid is the first eluted enantiomer. The study of chiral differentiation of alanine enantiomers by permethylated β-CD, using infrared multiple photon dissociation (IRMPD) spectroscopy combined with mass spectrometry, also showed the inclusion complex formation (Lee et al., 2017). However, the density functional theory calculations provided by Lee et al. (2017) for the lowest energy structures, were not consistent with those experimental results. The inclusion complexes formed by L-Ile zwitterions with both α- and β-CD showing 1:1 stoichiometry are confirmed by NMR (nuclear magnetic resonance techniques), surface tension and conductivity measurements (Roy et al., 2016).

Previously, we studied the inclusion complex formation and chiral discrimination of several amino acids with β-CD, by means of molecular mechanics (MM) and dynamics (MD) simulations (Alvira, 2013, 2015, 2017a, 2019). We have studied alanine (Ala), valine (Val), leucine (Leu), and isoleucine (Ile) in vacuo and with solvents like water, considering two configurations for the amino acids: non-polar and zwitterion. Furthermore, some improvements were made in the simulation method applied to each amino acid, concerning the calculation of molecular structures, parameters of interaction energies, or the number of trajectories in MD, among other factors. We showed that Ala, Val, Leu, and Ile were able to form inclusion complexes with β-CD in water and other solvents, but only Ile was included in vacuo. This diverged from the results proposed by Ramirez et al. (2000) and Lee et al. (2017). The most useful modification in the molecular simulation applied to Ile (Alvira, 2019) was the *ab initio* method used to determine the amino acid configurations, instead of the force field proposed by Weiner et al. for the molecular mechanics simulation of nucleic acids and proteins (Weiner et al., 1984, 1986), used in our earlier studies (Alvira, 2013, 2015, 2017a). The aim of the present work was to apply the model proposed for Ile to theoretically examine the interaction between Ala, Val, and Leu with β-CD in vacuo. To achieve this, the configuration of complexes formed by these molecules is determined by MM, as well as the differences in interaction energies between the enantiomers that cause the discrimination. Elution order, residence time, and capacity for inclusion complex formation are deduced from MD. The most important result and main difference with respect to our previous results is the capacity of Ala, Val, Leu, and Ile to form inclusion complexes with β-CD in vacuo. This is in agreement with the conclusions obtained by Ramirez et al. (2000), not only those related to inclusion complex formation, but also the lowest energy structure of the complexes and the elution order in the separation of these amino acid enantiomers.

## Materials and Methods

### Molecular Mechanics Simulation

The minimum energy complex structure is determined by MM simulation, and it is considered an inclusion complex if the guest (amino acid) is totally or partially inside the host (β-CD). The molecular configuration of β-CD and atomic charges are taken from the literature (Kinglert and Rihs, 1991), but the atomic coordinates of amino acids are calculated by the Hartree-Fock method, as previously for Ile (Alvira, 2019). The 6-31G^**^ basis set implemented in the MOLPRO package (Werner et al., 2012a,b, MOLPRO) is used to determine the amino acid structures, instead of the force field model proposed by Weiner et al. (1984, 1986) for nucleic acids and proteins. This is one of the improvements in the simulation method with respect to our previous studies (Alvira, 2013, 2015, 2017a, 2019). We considered an all-atom model for the molecules, as the H atoms can participate in H-bonds that can be important in structuring the complex. The interaction energy between the enantiomers and β-CD is calculated by locating the guest center of mass on a grid, and with different orientations (about 23,000) with respect to the host. The distance between two consecutive grid points is 0.1 Å, from −5 to 5 Å on each axis. The space-fixed reference system is located over the principal axis of β-CD, with the origin at the center of mass of the cavity and the XY plane perpendicular to the cavity axis. The guest orientation is defined by the Euler angles formed between the principal axis of the amino acids and the absolute reference system. The host-guest complex with the lowest energy is normally the complex configuration. The interaction energy *E* between β-CD and the amino acids (Ala, Val, Leu, and Ile) is determined as the sum of intermolecular *E*_inter_ and intramolecular *E*_intra_ terms, as in the AMBER force file proposed by Weiner et al. for molecular mechanics simulation of nucleic acids and proteins (Weiner et al., 1984, 1986). The van der Waals, electrostatic and H-bond energies contribute to *E*_inter_.

Amino acids are neutral molecules, but under some pH conditions or with polar solvents the amino acid appears as a zwitterion. The difference between the amino acid configurations (non-polar and zwitterion) are due to the presence of the $NH_{3}^{+}$ and *COO*^−^ groups instead of *NH*_2_ and *COOH*, which affects both the molecular structure and charge distribution. In our previous studies with amino acids, we analyzed the influence of various solvents (such as water) on the separation of enantiomers. To represent the solvent polarity, different values of ε were selected, as well as two atomic charge distributions and molecular structures for the amino acids. The presence of water in the separation process was simulated by ε = 80 and a zwitterion configuration for the enantiomers in those studies. The electrostatic contribution to the interaction energy between the amino acids and β-CD in the presence of water was about −0.4 kcal/mol, the discrimination between the enantiomers being due to the van der Waals contribution in this case. Residence times with water are generally shorter than in vacuo, although they depend on the amino acid enantiomer and initial conditions in the trajectories of MD (Alvira, 2013, 2015, 2017a, 2019). Ramirez et al. assumed a non-polar structure for the guest molecule and a dielectric constant of 1, in the molecular dynamics calculations of the inclusion complexes formed by some amino acids and β-CD in gas-phase (Ramirez et al., 2000). This was despite the fact that the authors started the experiment with a solution that was electrosprayed into the Fourier transform mass spectrometer (Ramirez et al., 2000). In the present study, we use the same conditions as Ramirez et al. to simulate the separation of some amino acid enantiomers by β-CD in vacuo (a non-polar structure and ε = 1). The H-bond term only contributes to *E*_inter_ when the complex configuration fits the distances and angles required to form this type of bond (O-H, N-H) (Weiner et al., 1984, 1986). *E*_intra_ represents the conformational adaptation of host and guest; it is the result of adding the torsional energy, bond stretching and bending functions.

The optimized parameters (ff99SB-ILDN) for the torsion potential of the AMBER ff99SB protein force field are used for Leu (N-C^α^ − *C*^β^ − *C*^γ^) and Ile (N-C^α^ − *C*^β^ − *C*^γ2^) (Lindorff-Larsen et al., 2010). This is another variation included in the present molecular modeling process, although its influence on the results and conclusions is not as important as the method used to calculate molecular structures, since *E*_intra_ is not very different for the enantiomers and its magnitude is usually smaller than *E*_inter_.

However, the enantioseparation is a dynamic process in which the amino acid is continually moving, and the complex does not always adopt the lowest energy configuration. Therefore, the different interaction energies between the enantiomers and β-CD (chiral discrimination) do not depend only on their minimum value. MM provides the minimum energies in the guest dispositions on the grid inside and outside the cavity (the potential energy surface, PES). The results of MM are also shown in the penetration potential W, which represents the minimum energy in each plane parallel to XY, against the cavity axis. The shape of W allows us to predict if the guest can be included more or less easily in β-CD or can even be repelled from the cavity, depending on whether W resembles a well or a barrier potential. Since the guest is not able to adopt the most energetically favorable disposition in each grid position, we also determined the PES in a different way. This involved calculating the average Boltzmann energy for the different orientations of the enantiomer instead of the minimum value. We are really interested in the capacity of β-CD to separate the amino acid enantiomers, therefore the difference in the PES is useful to identify the regions with greater chiral discrimination (greater differences in energy).

### Molecular Dynamics Simulation

The relative motion between host and guest is determined in MD by solving the classical equations of motion due to their mutual interaction. The solution cannot be obtained analytically but numerically, since there are many particles in the system, each with many constraints. To integrate the equations, we used an in-house computer program written in Fortran with the MD simulation performed at constant temperature (293 K). For this, a variant of the leap-frog scheme proposed by Brown and Clarke (1984) was used, in which the kinetic energies of rotational and translational motions are separately constrained (Fincham et al., 1986). Moreover, both types of movement are posed and solved in different equations, since the translation of the guest center of mass is due to the total force acting on the particle, and the rotation about this center of mass is a consequence of the total torque. The rotational equations of motion for the guest are posed as a function of four quaternions instead of angular variables, to avoid the problems of divergence. Therefore, these equations include quaternions, their first derivatives, and angular velocities and their time derivatives (Allen and Tildesley, 1987; Rapaport, 1995; Frenkel and Smit, 2002). In this way, MD reveals the evolution of the molecules, i.e., the variation in atomic positions and velocities through time (trajectory). However, the solution of the equations of motion, and therefore the resultant trajectory, depends on the initial conditions considered for the velocities (rotational and translational) and molecular dispositions. Therefore, some trajectories are determined in the simulation to reflect different situations the system can reach in the separation process. The magnitude of initial velocities is calculated consistently with the temperature at which the process occurs (293 K). Their direction is randomly computed; it is the same for all the amino acids, to avoid the simulation being influenced by factors other than the interaction with β-CD. However, the initial disposition of enantiomers in the trajectories cannot be the same because they are mirror images and, although there is no single method to select them, it is a question insufficiently dealt with in MD simulations. In our previous studies with amino acids, we tested different models to calculate the initial dispositions of enantiomers based on energetic, numerical or geometrical criteria (Alvira, 2017b). The method applied in the present study minimizes the differences in the van der Waals energy and average atomic distances between the enantiomers, as we already adopted in our more recent research with Ile (Alvira, 2019). The smallest differences in both energy and atomic positions are those of Val (average values 3.8 × 10^−6^ kcal/mol, 0.11 Å), very similar to Ile (1.6 × 10^−5^ kcal/mol, 0.14 Å), then Leu (2.8 × 10^−5^ kcal/mol, 0.40 Å). The smallest amino acid (Ala) has the greatest differences (3.7 × 10^−4^ kcal/mol, 0.96 Å). Twenty trajectories are calculated in the simulation, with initial enantiomer dispositions outside the cavity facing the rims of β-CD, since when the guest starts from positions near the cavity walls it does not enter the cavity but moves away. We consider different guest orientations parallel to the rims and pointing toward the wide or narrow rim of β-CD (Alvira, 2019), doing the same for all the amino acids to make the results comparable. The simulation time for each trajectory is 5 ns with a step of 1 fs, and the energies and guest dispositions were registered every 100 steps. However, the interaction between the amino acid and β-CD is weaker outside than inside the cavity, moreover it decreases as the distance between them increases, to such an extent that at some guest positions their interaction is not attractive enough to include the amino acid in β-CD. The molecule usually enters the cavity where it stays for some period of time, moving continually and then leaving the CD. However, there are some trajectories in which the amino acid cannot be included totally or partially in the cavity (external trajectories). The number of external trajectories for each amino acid is different despite the initial conditions being the same for all of them, and it does not depend on the initial dispositions of the enantiomers. We integrate the equations of motion during the period of time the guest remains near the cavity, both inside the cavity (residence time) or outside for the external trajectories. To locate the most probable position of the enantiomers under the influence of β-CD, we determine the position probability density as the number densities of guest positions in a volume element, divided by the total number of guest positions in the simulation (Lipkowitz et al., 1997a,b). The number density is obtained from a grid in which the distance between two consecutive points is 0.5 Å. The position probability density reflects the external trajectories and indicates the capacity to form inclusion complexes, since it represents the most probable positions of the guest center of mass. The enantiomers are moving continually, but they also have preferential orientations when they are in the preferred locations, these dispositions being the most probable configurations of the complexes deduced from MD. Whereas, the position probability density indicates the capacity to form inclusion complexes, the enantioselectivity is provided by the difference in the binding free energy *F* of the enantiomers. *F* indicates if the complex is more energetically favorable than the reactants and is calculated in each trajectory (Rapaport, 1995). The energy in *F* is calculated as the sum of intermolecular and intramolecular terms, this way the effect of the conformational adaptation of host and guest is also included in the results: binding free energies, elution order, residence times, position probability densities and the most probable configuration of complexes. The elution order is determined by the difference in *F*_*mean*_ between the enantiomers Δ*F*_*mean*_, where *F*_*mean*_ is the average binding free energy in the simulation. If Δ*F*_*mean*_ > 0, the D-enantiomer of amino acids is more tightly bound and the L-enantiomer is eluted first. The MD model used in the present research was previously applied in our study with Ile, but it includes some improvements on the simulations carried out with Ala, Val and Leu, regarding the number of trajectories (6 for Ala and Val, 12 for Leu), simulation time (3 ns) and the most influential initial enantiomer dispositions.

## Results and Discussion

### Molecular Mechanics Simulation

**Figure 1:**
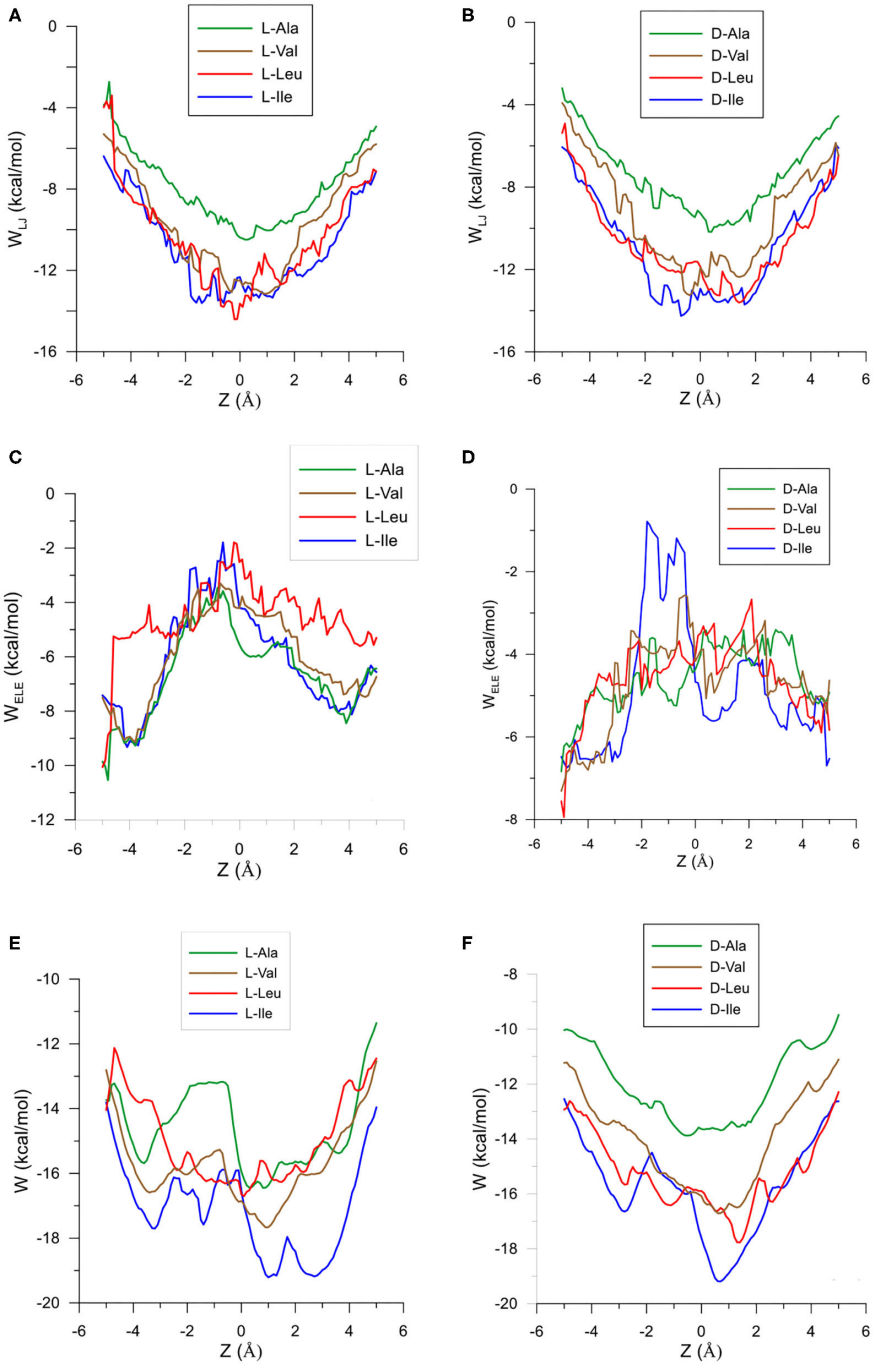
The Lennard-Jones contribution to the penetration potential W_LJ_ (kcal/mol) for the interaction between β-CD and **(A)** L-enantiomers of the amino acids, **(B)** D-enantiomers of the amino acids. The ELE contribution to the penetration potential W_ELE_ (kcal/mol) for the interaction between β-CD and **(C)** L-enantiomers of the amino acids, **(D)** D-enantiomers of the amino acids. The penetration potential W for the interaction between β-CD and **(E)** L-enantiomers of the amino acids, **(F)** D-enantiomers of the amino acids.

**Figure 2:**
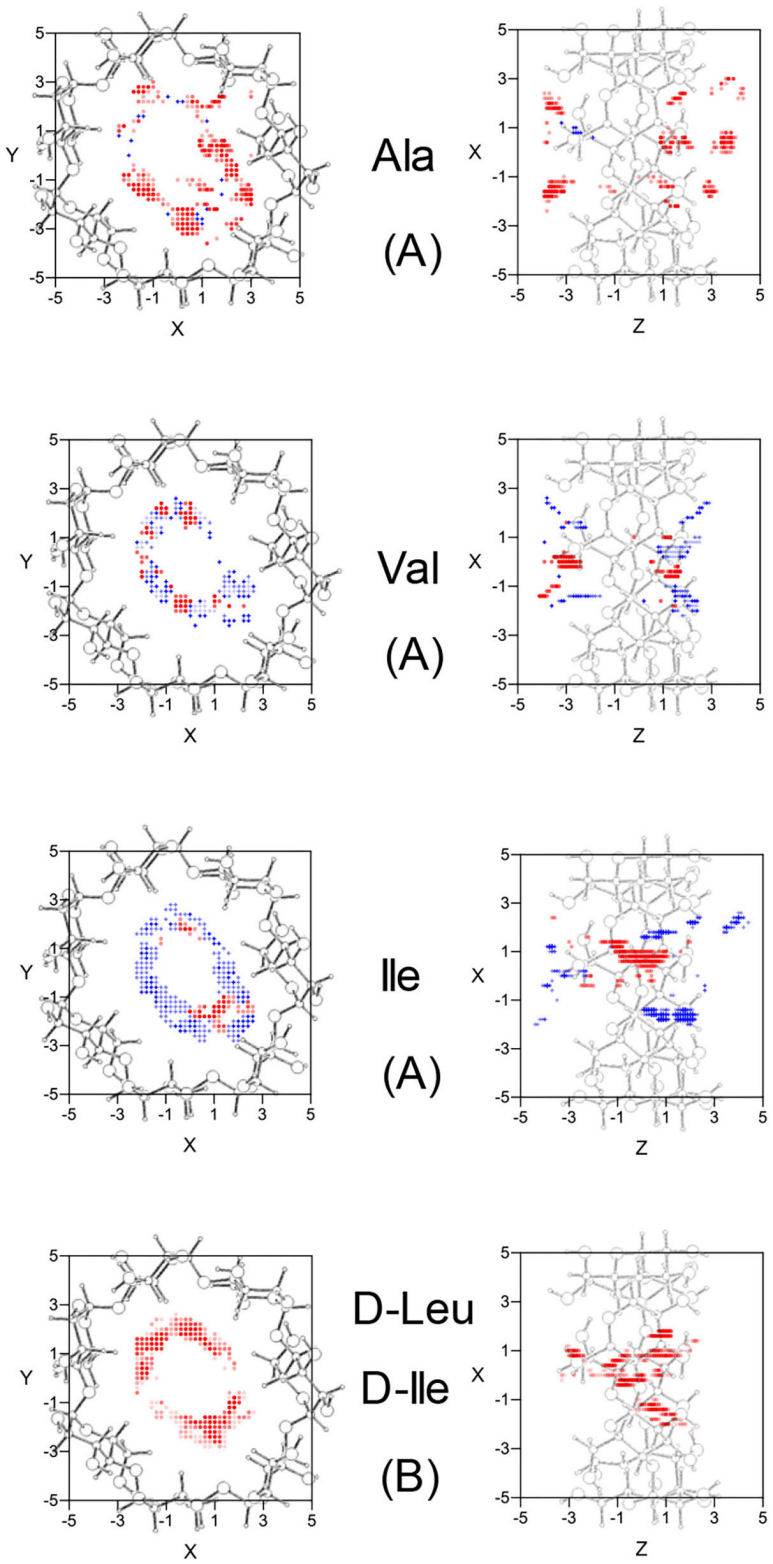
**(A)** The projections in the XY and XZ planes of the potentially most discriminating regions for the amino acid enantiomers of Ala, Val, and Ile. The zones where the L-enantiomers are more stable are represented by red circles, those for D-enantiomers by blue crosses, and the more intense the symbol the greater difference in energy it represents. **(B)** The projections in the XY and XZ planes of the potentially most discriminating regions for the isomers D-Leu and D-Ile. The zones where D-Ile is more stable are represented by red circles, those for D-Leu by blue crosses, and the more intense the symbol the greater difference in energy it represents. A schematic representation is included of the projections of β-CD in those planes.

The configuration of complexes formed by the amino acids and β-CD is obtained in MM by the guest orientation and position with respect to the cavity, for which the interaction energy is the lowest *E*_min_. To determine this absolute minimum, the energy *E* must first be calculated in each guest position inside and outside the CD for different orientations of amino acids. The minimum result of *E* is assigned at each grid point. The potential energy surface (PES) represents these minima for the different dispositions of amino acids with respect to β-CD, the energy *E* being determined by the sum of inter- and intramolecular terms. The intermolecular energy *E*_inter_ is calculated by adding the Lennard-Jones (LJ), electrostatic (ELE) and H-bonds contributions, and the penetration potential W represents the consecutive minima of *E*_inter_ in each plane perpendicular to the cavity axis. This W allows us to visualize if the guest can enter or is repelled by the CD, depending on whether it resembles a well or barrier potential. The main contribution to W is due to the Lennard-Jones term (W_LJ_), which appears as a well potential for the enantiomers (Figures 1A,B) and therefore contributes to the inclusion of amino acids into the cavity. The larger amino acid is the deeper W_LJ_, and its sharpened shape is due to the difficulty for the guest to orientate freely inside the cavity. The difference in W_LJ_ between the enantiomers of an amino acid (Figures 1A,B) also increases with the molecular size, and corresponds to inner more than outer guest positions. For the isomers Leu and Ile, W_LJ_ differs more for D- than L-enantiomers and occurs when the molecule is near the narrow rim of β-CD. The H-bond energy only contributes to *E*_inter_ in those guest dispositions that fit the conditions needed to form H bonds, between the O and H atoms of amino acids and CD (Weiner et al., 1984, 1986). The maximum value of this term is −0.5 kcal/mol, similar for all the amino acids, although its influence in *E*_inter_ is different due to the percentage it amounts to for each one. Whereas, W_LJ_ appears as a well potential, the electrostatic contribution to W (W_ELE_) for the amino acids seems a barrier potential because its minimum is located outside the CD, and it increases inside the cavity (Figures 1C,D). W_ELE_ is very similar for the L-enantiomers, both the extreme values (minimum and maximum) and the location of the greatest value near the cavity center (Figure 1C). However, the ELE energy is very different for the D-enantiomers in shape and magnitude. The maximum value of W_ELE_ inside the cavity corresponds to D-Ile and is located near the narrow rim of β-CD, while the greatest ELE energy for D-Ala and D-Leu occurs near the wide rim (Figure 1D). This term can reach 30 % of W, and the differences in W_ELE_ between the amino acid enantiomers inside the cavity increases with the molecular size, from 1.40% (for Ala) to 78.09% (for Ile). In fact, their discrimination is due mainly to this term, as deduced by comparing the curves W_LJ_ (Figures 1A,B) and W (Figures 1E,F). The ELE is also directly related to a barrier potential in W for some amino acids, between the narrow rim and the center of the cavity. It is also related to the guest orientation with respect to the cavity axis. The height of this barrier is similar for L-Ala, L-Val, L-Ile, and D-Ile, but the width decreases as the size of the guest increases. Isoleucine is the most stable amino acid and its enantiomers present the lowest differences in W, both in shape (potential barrier) and magnitude. To represent the PES, the interaction energy *E* in each grid point is determined as the average Boltzmann energy corresponding to different guest orientations, instead of the minimum value. This is because the molecule is not always able to adopt the most stable configuration in the separation process. Figure 2A shows the projections of *E*_*L*_−*E*_*D*_ in the XY and XZ planes, *E*_*L*_ and *E*_*D*_ being the energies for each enantiomer of Ala, Val and Ile. A schematic representation of the projections of β-CD in those planes is included. The positions at which *E*_*L*_−*E*_*D*_ < 0 are represented by red circles and blue crosses denote the most stable positions for the D enantiomer. The more intense the symbol, the greater difference in energy it represents. The potentially most discriminating regions for all of the amino acids are located near the cavity walls, where the interaction energy changes from negative to positive values. However, the stability of enantiomers and the zones where the differences in energy are greater depend on the size of the amino acids. Whereas, L-Ala is the most stable enantiomer in broader zones of PES, D-Ile frequently has lower energies. Moreover, the regions with greater chiral discrimination are located in inner positions as the size of the amino acids increases, so L-Ala is more stable outside the CD cavity while L-Ile is more stable inside. Isoleucine is also more stable than Leu at every point of the potential surface; the greatest differences are found at the inner positions (Figure 2B).

The absolute minimum interaction energy *E*_min_, the values for the enantiomers and their different contributions are included in Table 1. The complexes formed by β-CD with Val are the most stable while Leu is the least. The Lennard-Jones term makes the greatest contribution to *E*_min_ for the amino acids, and the energy due to the angle bending *E*_angle_ contributes most to the intramolecular energy *E*_intra_. The bond lengths, bond angles and torsional angles of amino acids that contribute most to *E*_intra_ is different for the enantiomers, they are related to the atoms of guests located nearer the cavity walls in the complexes formed with β-CD. However, the average variations in angles and bond lengths due to the conformational adaptation of host and guest, is the order of 10^−4^ (degrees) and 10^−5^ Å, independently of the size of amino acids. The difference between enantiomers increases in order Ile < Leu < Val < Ala, being due mainly to the van der Waals energy except for Leu, in which it is caused by the electrostatic energy.

**Table 1:** The minimum interaction energy *E*_min_ obtained with the AMBER force field for each enantiomer and the different contributions: Lennard-Jones *E*_LJ_, electrostatic *E*_ele_, hydrogen bonding *E*_H-bond_, bond stretching *E*_bond_, angle bending *E*_angle_, and torsion energy *E*_torsion_.

**Amino acid**	***E***_**min**_ **(kcal/mol)**		***E***_**LJ**_ **(kcal/mol)**		***E***_**ele**_ **(kcal/mol)**		***E***_**H-bond**_ **(kcal/mol)**		***E***_**bond**_ **(kcal/mol)**		***E***_**angle**_ **(kcal/mol)**		***E***_**torsion**_ **(kcal/mol)**	
	**L**	**D**	**L**	**D**	**L**	**D**	**L**	**D**	**L**	**D**	**L**	**D**	**L**	**D**
Ala	−11.51	−9.04	−10.83	−8.95	−5.85	−4.98	0.00	0.00	1.59	1.80	3.42	3.03	0.16	0.06
Val	−11.08	−11.66	−13.14	−11.65	−4.55	−4.73	0.00	−0.44	1.54	1.42	4.32	3.04	0.75	0.70
Leu	−7.94	−8.19	−13.79	−13.59	−2.56	−3.79	−0.49	−0.46	1.42	1.43	4.71	4.97	2.77	3.25
Ile	−10.91	−10.82	−11.89	−13.60	−7.38	−5.67	0.00	0.00	1.45	1.52	4.76	4.81	2.15	2.12

**Figure 3:**
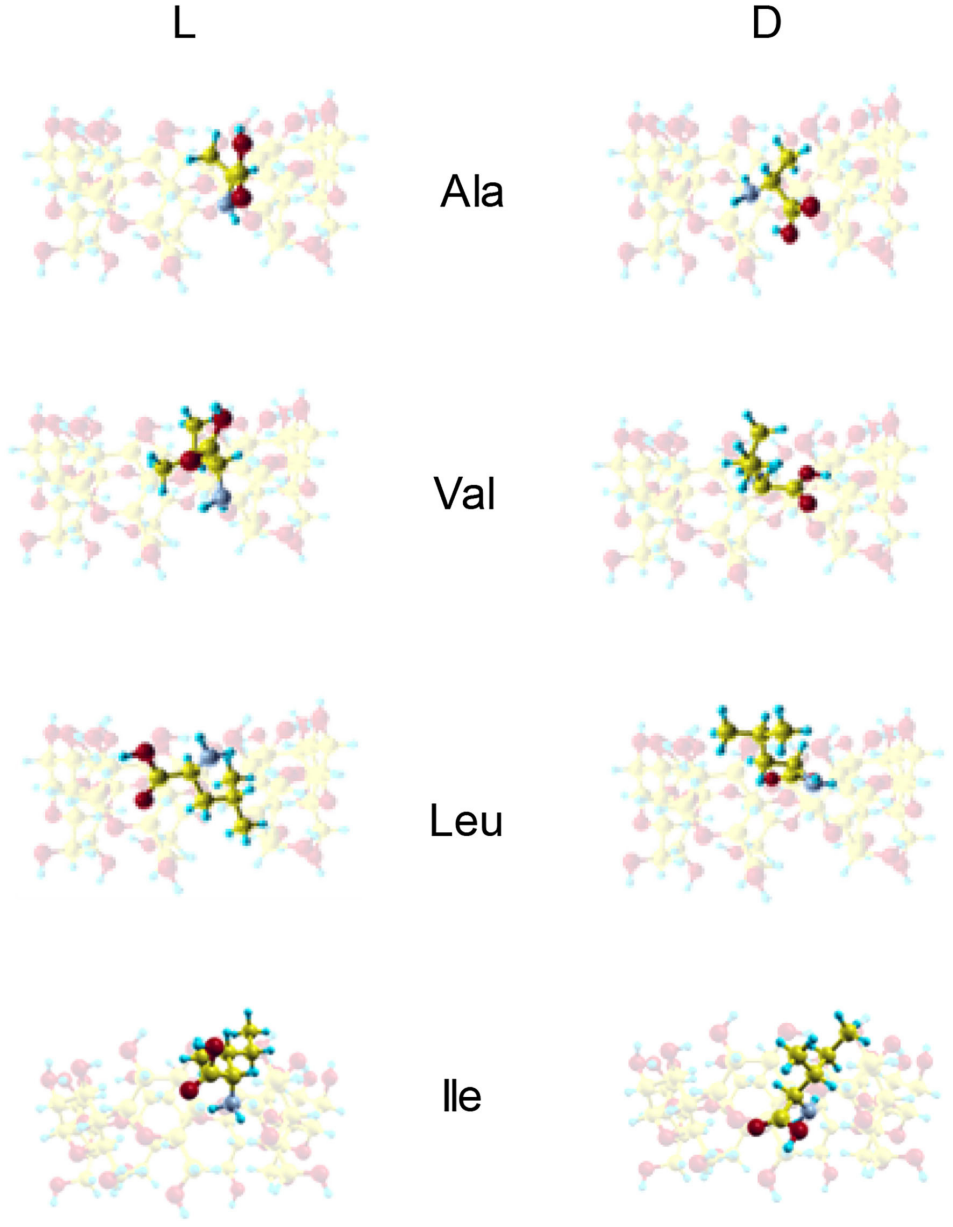
The configurations of the complexes with minimum interaction energy formed by β-CD and the amino acids in vacuo obtained from molecular mechanics. The guest molecule has been superimposed for clarity, but it is always located inside the cavity.

Figure 3 shows the configurations of the complexes with *E*_min_ formed in vacuo by β-CD with the amino acids (Kokalj, 2003). The guest molecule has been superimposed in Figure 3 for clarity, but they are inclusion complexes because the guest is totally or partially inside the cavity. D-Ala, D-Val and L-Leu are located near the cavity center in the complexes formed with β-CD, while L-Ala, L-Val and the enantiomers of Ile are near the cavity walls (Table 2). In these complexes, the amino acids are oriented in such a manner as to include the greatest part of the molecule, and therefore to achieve the maximum interaction with the CD. D-Leu and L-Ile are nearly parallel to the wide rim of the cavity, and this is due to the location of their center of mass. The guest orientation is different for the enantiomers: whereas the carboxylic end of L-amino acids is nearer the wide rim of CD, being oriented toward the narrow rim for D-amino acids. If we consider the unit vector defined by the chiral center of amino acids and the most distant C atom of the chain, the angles in spherical coordinates (θ, φ) formed by the enantiomers with respect to the absolute reference system are shown in Table 2. The configurations of L-Val, D-Ile and L- and D-Leu agree with the results of Ramirez et al. (2000) regarding inclusion complex formation, and the lowest energy structure of complexes in gas-phase.

**Table 2:** The guest center of mass positions and angles in the configurations of the complexes with *E*_min_ formed in vacuo by β-CD with the amino acids.

**Amino acids**	**Center of mass (x, y, z)**		**Angles** **(θ, φ)**	
	**L**	**D**	**L**	**D**
Ala	(0.6, −1.5, 0.3)	(0.7, −0.7, −0.6)	(51.80°, 63.63°)	(49.16°, 315.90°)
Val	(0.1, −1.2, 0.9)	(0.0, −0.5, 0.7)	(38.96°, 125.95°)	(46.72°, 20.18°)
Leu	(−0.6, −0.2, 0.1)	(−0.4, −0.1, 1.4)	(94.21°, 321.21°)	(61.80°, 168.17°)
Ile	(1.7, −0.4, 2.7)	(1.1, −0.9, 0.7)	(69.68°, 130.50°)	(43.29°, 270.93°)

### Molecular Dynamics Simulation

To determine the elution order, retention time and capacity for inclusion complex formation in MD, some trajectories (20) are calculated, starting from different initial conditions. In each trajectory, the molecule usually moves toward the β-CD and only in a few cases does not enter the cavity and remain a short time near β-CD (external trajectories). In most cases, the guest enters the CD, where it remains totally or partially during some period of time (residence time) and then moves away. The external trajectories have been excluded from the calculations in the simulation, because the utility of CDs for catalysis and separation processes is based on their capacity to form inclusion complexes. The initial disposition of enantiomers influences the evolution of guests in the trajectories and then the binding free energy and elution order. Therefore, the starting positions and orientations of enantiomers must be selected in such a way that the differences in their simulation were mainly due to their mirror image structures, instead of the calculation method. In the present study, the starting dispositions are determined by minimizing the difference in atomic positions and interaction energies. Furthermore, to compare the results of different amino acids, the same initial conditions (configurations and velocities) are considered. The evolution of enantiomers in each trajectory is due both to the initial disposition and molecular characteristics. In fact, only L-Ala and D-Val have not external trajectories, in spite of the same initial conditions for the amino acids. The probability of remaining outside the cavity is greater when the guest starts from dispositions near the narrow rim, with the carboxylic end pointing toward the cavity.

**Figure 4:**
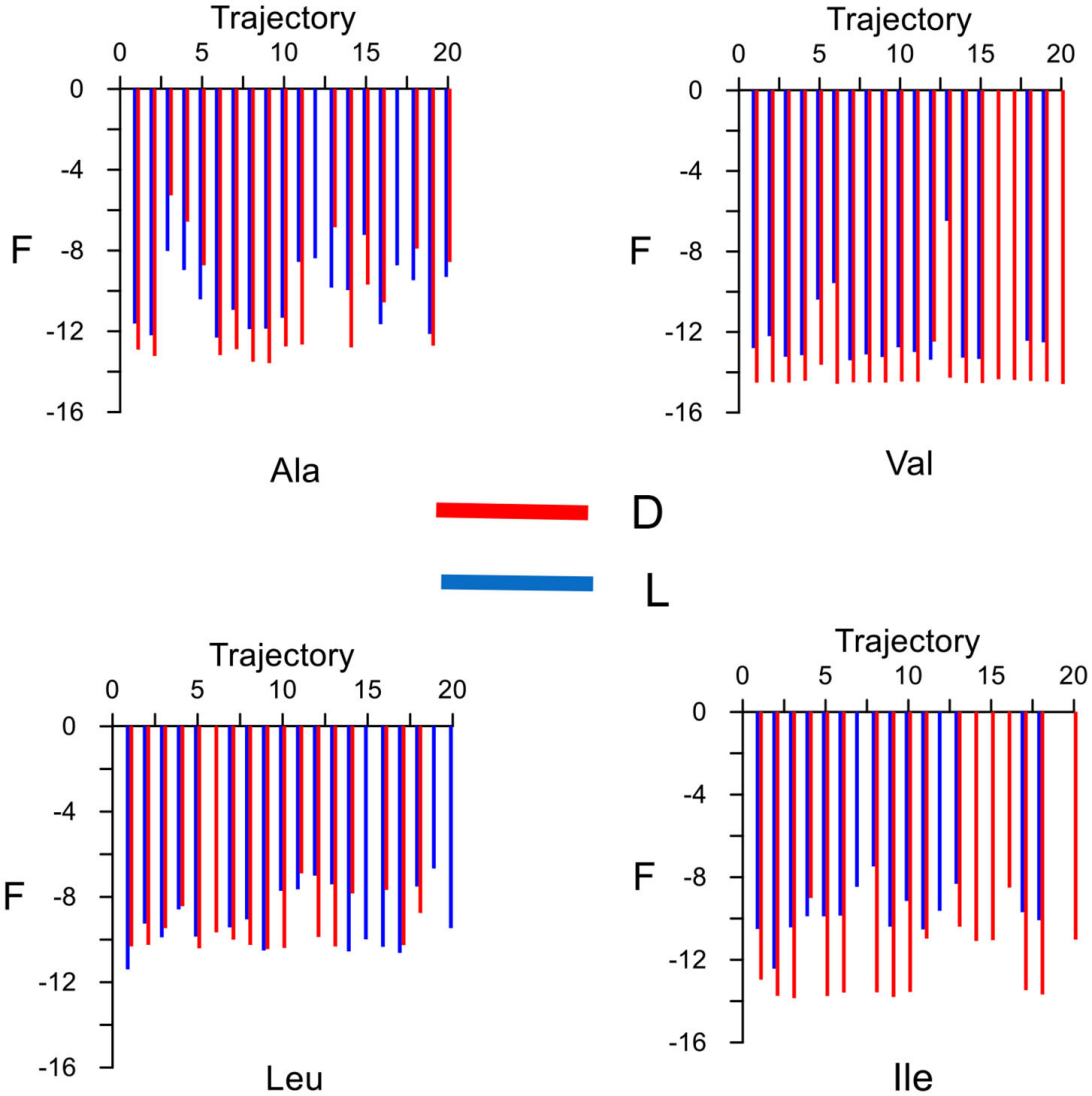
The average binding free energy for each amino acid derived from the trajectories in the molecular dynamics simulation. The external trajectories are excluded.

The elution order is determined from the average binding free energy, which is obtained for each trajectory *F* in the simulation. The results are shown in Figure 4 except for external trajectories (Table 3). The values of *F* are substantially lower for inner than outer trajectories, where *F* can even become positive. These results confirm that the ability of CDs to perform catalysis and separation processes is due to their inner cavity. The same initial conditions produce different energies *F* for each molecule because *F* is related to the guest evolution in the trajectories, which is due in turn to the molecular structure and composition. The binding free energy for each amino acid in the simulation *F*_*mean*_ is determined as the average value of *F*, considered only the inner trajectories (Table 3). D-Valine is the amino acid with the lowest energy *F*_*mean*_, while L-Leu has the greatest value, moreover *F* is lower for D- than L-enantiomer of Val and Ile in every trajectory. The difference in *F*_*mean*_ for the enantiomers Δ*F*_*mean*_ of each amino acid increases in the order Leu < Ala < Val < Ile, so if the capacity of β-CD to discriminate is related to this magnitude, Ile exerts the clearest enantiodiscrimination. The elution order is obtained from Δ*F*_*mean*_, the molecule least bound to β-CD (greater *F*_*mean*_) spends less time inside the cavity. The first eluted enantiomer in vacuo for each guest is the L-amino acid, in agreement with the experimental findings and molecular simulation provided by Ramirez et al. (2000). The present study is also in accordance with theirs in that the chiral discrimination increases with the size of amino acids, except in the case of Leu, for which they obtained the same selectivity as Ile. The average residence time *t*_mean_ for each amino acid in the simulation (Table 3) is not directly related to *F*_*mean*_, as can be deduced from the fact that Leu has greater residence times but lesser free energy. This question can be justified because *F*_*mean*_ depends on the interaction energy with β-CD and then on the guest dispositions during the trajectories. However, the amino acids are not always able to adopt the orientation with minimum energy in each location. Therefore, the guest can stay totally or partially inside the cavity but with configurations other than the minima. The greatest difference in *t*_mean_ between enantiomers is that for Ile, in accordance with Δ*F*_*mean*_, then Leu, Ala and finally Val. However, the new feature introduced by the present study is that the first eluted enantiomers also spend shorter times inside the cavity. The agreement between the elution order and *t*_mean_ was not achieved in our previous studies of amino acids, and this issue also confirms the improvement of the simulation method applied in the present research.

**Table 3:** The average binding free energy *F*_*mean*_, elution order and average residence time *t*_*mean*_ obtained for each enantiomer in the molecular dynamics simulation.

**Amino acids**	**Number of trajectories**		***F***_*****mean*****_**(kcal/mol)**		**Elution order**	***t***_*****mean*****_ **(ps)**	
	**L**	**D**	**L**	**D**		**L**	**D**
Ala	20	18	−10.23 ± 1.56	−10.78 ± 2.72	L	476.27	1683.71
Val	17	20	−12.24 ± 1.77	−14.32 ± 0.47	L	3400.55	4484.07
Leu	19	17	−9.09 ± 1.37	−9.47 ± 1.13	L	1682.58	3165.90
Ile	15	17	−9.77 ± 1.11	−12.22 ± 1.76	L	751.13	4582.92

**Figure 5:**
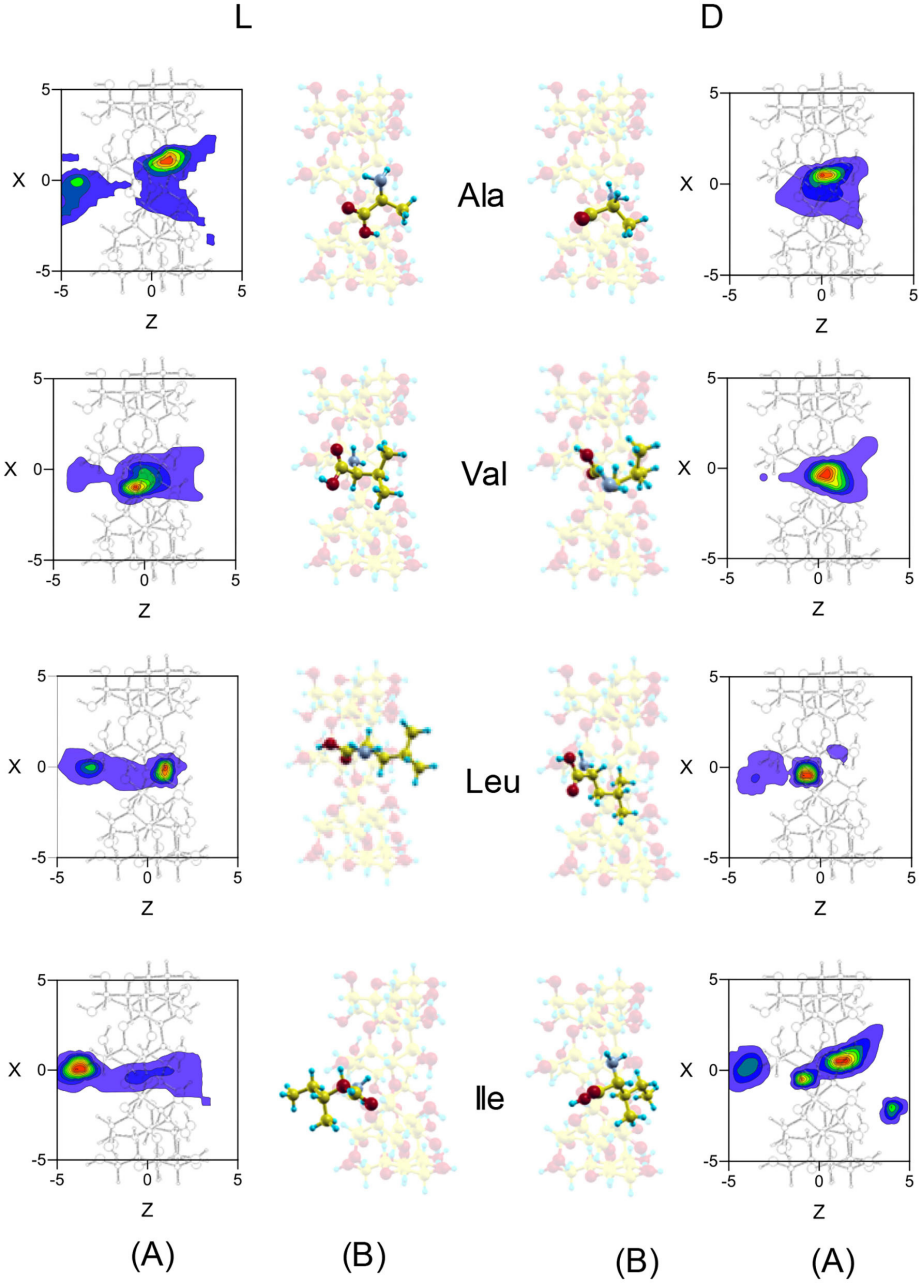
**(A)** The projections in the XZ plane of the position probability densities for the enantiomers of amino acids obtained from the molecular dynamics simulation. A schematic representation is included of the projection of β-CD in that plane. **(B)** The most probable configurations of the complexes formed by β-CD and the amino acids in vacuo, obtained from the molecular dynamics simulation. The guest molecule has been superimposed for clarity, but it is always located inside the cavity.

Both *F*_*mean*_ and *t*_*mean*_ indicate that the complexes formed by β-CD and the amino acids are inclusion complexes, as predicted by other authors (Ramirez et al., 2000; Lee et al., 2017), and the most probable configurations in MD are deduced from the position probability density (Lipkowitz et al., 1997a,b). The location where the guest center of mass spends most time in the simulation is the center of the cavity for Ala and Val, but Leu and Ile remain near the wide rim (L-Leu, D-Ile) or the narrow rim (D-Ile, L-Ile) of β-CD (Figure 5). The capacity of guest molecules to move freely within the CD is related to their size and structure, among other factors. Ala and Val can move through the cavity and then the preferred center of mass position does not depend on the initial disposition of amino acids. The most probable zones for the guest in the inclusion complexes formed by Ala and Val are stable positions with lower interaction energies and also slightly enantioselective regions. The molecule is rotating continually along the trajectories, but the most frequent guest orientation for Ala and Val locates the carboxylic end pointing toward the narrow rim of β-CD (Figure 5) (Kokalj, 2003). The guest molecule has been superimposed in Figure 5 for clarity, but it is always located inside the cavity. The configurations of D-enantiomers are similar to the absolute minima energies, and only L-Val is in accordance with that proposed by Ramirez et al. (2000). There are two regions where Leu and Ile remain more time in the simulation, near each rim of β-CD. This can be explained by the existence of a barrier potential in the energy W, related to the molecular orientation with respect to the cavity axis. Alanine and Val remain in zones that are not so enantioselective, whereas Leu and Ile frequent regions with great chiral discrimination. Since Leu and Ile are unable to rotate freely inside the cavity, their evolution through the simulation depends on their initial orientation and which rim of CD they approach from Alvira (2019). The greater residence times for Leu and Ile correspond to trajectories with initial dispositions of the amino acid near the wide rim and with the amino end pointing to β-CD. However, in the preferred locations of these amino acids, the carboxylic end is near the narrow rim of the cavity (Figure 5) (Kokalj, 2003). The most probable configuration of the complexes formed by Ile and β-CD can explain their clearest enantiodiscrimination, since this amino acid possesses two chiral centers, whose interactions with β-CD contribute more significantly to the energy. Therefore, whereas only one chiral center of L-Ile is near the cavity, the two chiral centers of D-Ile are located inside, increasing the difference in energy with L-Ile. The host-guest complexes formed by D-Leu and D-Ile are consistent with those proposed by Ramirez et al. (2000), and the configuration of D-Ile is also similar to that of minimum energy obtained by MM.

## Conclusions

The inclusion complexes formed by β-cyclodextrin and some amino acids in vacuo, as well as the chiral separation of these enantiomers, are simulated in this study by molecular mechanics and dynamics. The greatest contribution to the interaction energy obtained from MM is the van der Waals term, although the discrimination between the enantiomers is due mainly to the electrostatic energy. The configurations of absolute minimum energy for the amino acids correspond to inclusion complexes, in which the carboxylic end is near the wide rim of β-CD for L-enantiomers and pointing toward the narrow rim for D-enantiomers. The differences in the potential energy surfaces indicate the more enantioselective regions, which are located near the cavity walls in every case. However, the zones where each enantiomer is more stable depend on the size of amino acids: while L-Ala is more stable in wider zones outside the CD, L-Ile has lower energies inside the cavity.

The elution order, capacity to form inclusion complexes, and residence times are determined in MD by the calculation of some trajectories with different initial conditions. The elution order obtained from the average binding free energy indicates that L- is the first eluted enantiomer of amino acids, and the chiral discrimination increases in the order Leu < Ala < Val < Ile. The position probability density indicates the formation of inclusion complexes whose configurations are similar for the amino acids, inside and along the cavity axis, with the carboxylic end pointing toward the narrow rim of β-CD. During the simulation, Ala and Val remain in zones that are not so enantioselective, whereas Leu and Ile occupy regions with great chiral discrimination. Some of the results from the present research are in agreement with those proposed by Ramirez et al.: the lowest energy structure of complexes obtained from MM for L-Val, D-Ile, and L- and D-Leu; the elution order, inclusion complex formation, and the dependence of selectivity on the size of amino acids (except Leu) from MD. The improvement in the simulation method can be confirmed by the results obtained for Ala, Val, Leu, and Ile in vacuo, completely different from our previous results. However, the model proposed must be tested by new studies with more amino acids and other types of molecules.

## Data Availability Statement

All datasets generated for this study are included in the article/supplementary material.

## Author Contributions

The author confirms being the sole contributor of this work and has approved it for publication.

## Conflict of Interest

The author declares that the research was conducted in the absence of any commercial or financial relationships that could be construed as a potential conflict of interest.
